# False Negative Urine Pregnancy Test: Hook Effect Revealed

**DOI:** 10.7759/cureus.22779

**Published:** 2022-03-02

**Authors:** Shista Priyadarshini, FNU Manas, Sheela Prabhu

**Affiliations:** 1 Internal Medicine, Guthrie Robert Packer Hospital, Sayre, USA

**Keywords:** false negative poc upt, false negative test in late pregnancy, false negative upt, hook effect, false negative urine pregnancy test

## Abstract

The urine pregnancy kit tests are commonly used in women of childbearing age to detect pregnancy. However, these tests may fail to detect pregnancy, rarely leading to inadvertent lab and radiation exposure. The hook-effect is a rare but important phenomenon, rendering the kit tests false negative due to an improper antigen-antibody ratio.

## Introduction

Every time a woman of child-bearing age is admitted to a hospital, it is a rule of thumb to check for pregnancy. Ruling out pregnancy is of the essence to avoid any inadvertent radiation exposure lest fetal teratogenicity. With the advent of urine pregnancy kit tests in the 1970s [1], they have become an indispensable part of our healthcare system. The current system relies heavily on lab tests, imaging, and technology, making it inconceivable to imagine a day without them. However, we tend to lose our skepticism that these tests can sometimes be inaccurate and rate them above a well-performed history and physical examination. This manuscript aims to highlight a case that evaded diagnosis at multiple checkpoints, creating a false sense of security leading to a potential adverse health care event.

## Case presentation

A 33-year-old Caucasian woman presented to the emergency room with severe low back pain, aggravated with minimal exertion. She had a generalized limited range of movement and was unable to lie flat on her back. She had a past medical history of intravenous drug abuse (IVDU), tricuspid valve regurgitation, and methicillin-resistant *Staphylococcus aureus* (MRSA) endocarditis with valve replacement. She had two prior normal deliveries, a BMI of 21, and reported recent irregular menstruation (oligomenorrhea). She recently started using IV heroin and methamphetamine again. She was tachycardic but afebrile. On examination, she had severe tenderness in her lumbar spine and suprapubic fullness but without abdominal/costovertebral angle tenderness. Laboratory investigations showed elevated inflammatory markers (erythrocyte sedimentation rate (ESR), C-reactive protein (CRP)), normal amylase, and an unremarkable point-of-care urine pregnancy test (POC UPT). With concern for lumbar osteomyelitis and abscess in the setting of IVDU, she underwent CT abdomen and pelvis with contrast that showed L1-L2 acute discitis/osteomyelitis. In addition, 18 weeks of intrauterine pregnancy were found as well. The patient was unaware of the pregnancy.

## Discussion

Human chorionic gonadotropin (hCG) is a hormone produced by the trophoblastic tissue that helps in the development of the embryo and fetal placenta [2]. It is a heterodimeric glycoprotein consisting of alpha and beta subunits. After undergoing metabolism in the liver and further breakdown in the kidneys, it presents as a beta core fragment to be measured by the POC UPT kits [3-6].

The urinary kits target the beta subunit to avoid any confounding, as the alpha subunit is shared by other members of the glycoprotein family, including follicle-stimulating hormone (FSH), luteinizing hormone (LH), and thyroid-stimulating hormone (TSH). The point-of-care urine pregnancy kits are known to detect pregnancy as early as the first day of missed period by qualitative hCG hormone in urine. UPT is known to have a maximum sensitivity of 90% at this stage. Most kits detect the beta subunit of hCG in the range of 25 to 50 mIU/ml, whereas others range from 15 to 100 mIU/ml [7]. 

Many studies have documented the presence of variants of hCG in early pregnancy urine [8], including intact hCG, free beta chain, beta core fragment, nicked free beta, nicked hCG, hyperglycosylated hCG, etc [9]. Different pregnancy kits have different sensitivity levels based on the duration of gestation or the quantity and quality of hCG. The urinary pregnancy kits usually have mono or polyclonal antibodies to attach to the most common variant, “intact hCG” [10].

**Figure 1:**
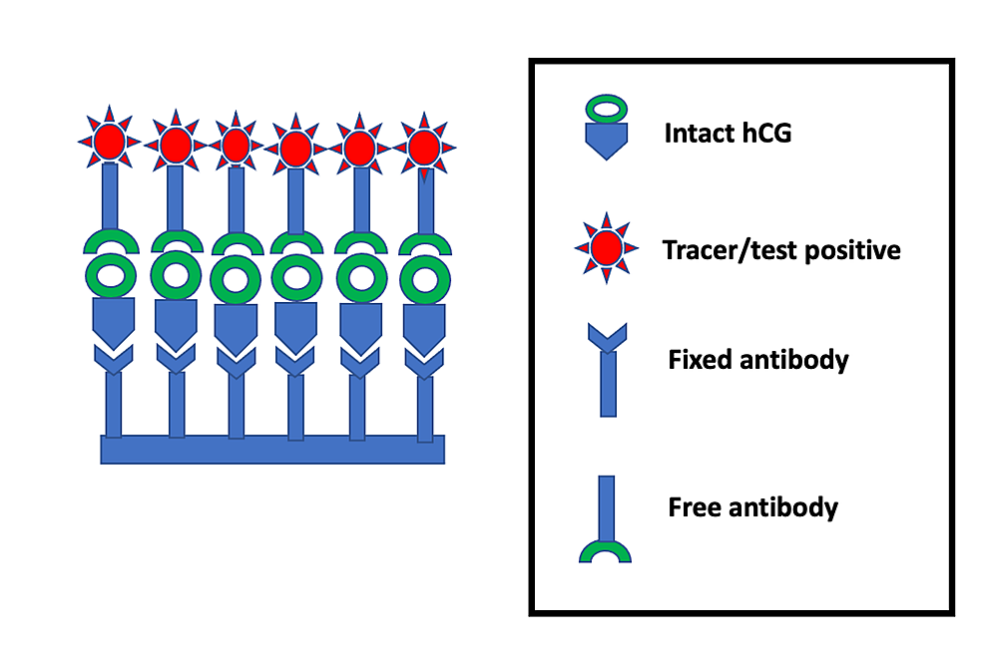
Functioning of a normal urine pregnancy test (UPT). hCG: human chorionic gonadotropin.

As depicted in Figure 1, the kits consist of a fixed antibody attached to a base and a free antibody attached to a tracer. Once these fixed and free antibodies bind to hCG, a complex is formed and the tracer lights up, rendering the test positive. As handy as it may seem, there are numerous factors involved in the analysis of these urinary pregnancy kits.

**Figure 2:**
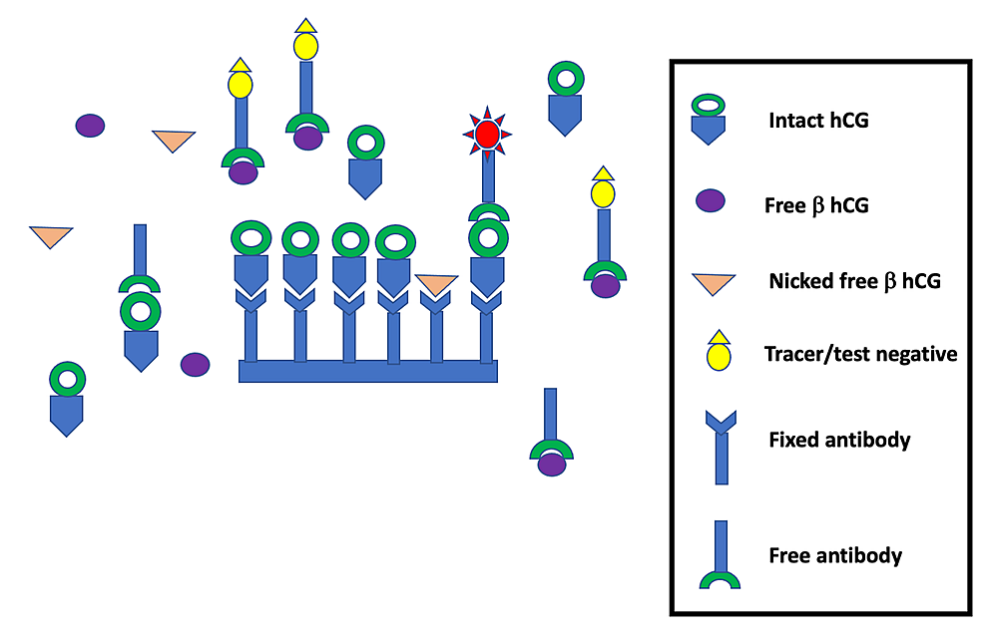
Incomplete complex formation presenting as false negative UPT. UPT: urine pregnancy test, hCG: human chorionic gonadotropin.

A false negative test results, when the uncommon variants such as free beta chain, nicked free beta, and beta core fragments (monomer) are the predominant components in some pregnancies [11]. In these situations (Figure 2), as the heterodimeric form of hCG is not present, the antibodies in the kit are unable to form a complex and manifest the test as negative.

**Figure 3:**
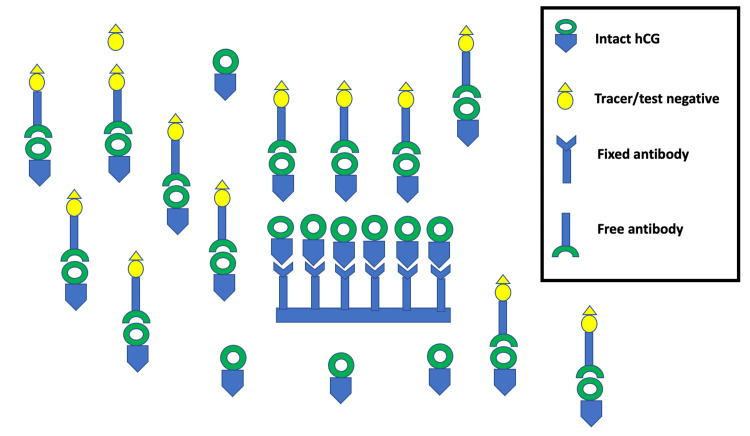
Excess antibodies leading to incomplete complex formation presenting as false negative UPT. UPT: urine pregnancy test, hCG: human chorionic gonadotropin.

In this case, the pregnancy kit was falsely negative, while the CT abdomen and pelvis with contrast revealed an 18-week pregnancy. As mentioned before, the hCG levels of 15 to 100 mIU/ml are usually targeted by pregnancy kits, which implies that levels lower than these are not likely to be detected. On the other hand, the hCG levels start rising gradually with increasing gestational age and are >100 mIU/ml from four weeks onwards [12]. As in our case, once the hCG levels are elevated with advancing pregnancy, a prozone phenomenon is observed. This phenomenon is due to excess hCG antigen when compared to the finite antibodies present in the pregnancy kits. With the antigen being in excess, the formation of complex/sandwich is hindered and is reflected as falsely negative, as shown in Figure 3. This phenomenon is also known as the “hook effect.”

Interestingly, urine dilution prior to repeating the kit test often overcomes the “hook effect” as it reduces the antigen: antibody ratio. This manuscript aims to educate healthcare personnel about the rare but important “hook effect,” especially when the clinical picture is not congruent with the lab test. This case describes a scenario where pregnancy was missed because of inconsistent history, irregular menstruation, the patient's being unaware of her pregnancy and the results of her pregnancy test being negative as a result of "hook effect." Similar to our patient, there could be instances, as mentioned above, that can make the UPT false negative, so it should be kept in mind. In such scenarios, the tests should be repeated with diluted urine to balance the excess antigen or with different UPT kits to target the uncommon antigens.

## Conclusions

To sum up, this manuscript highlights a rare phenomenon associated with the very commonly used urine pregnancy tests (UPTs). In late pregnancy, with the abundance of hCG antigens in-vivo, the antigen: antibody ratio required for the test to be positive is hampered. This rare phenomenon is known as the “hook effect.” This results in the negative pregnancy test and can lead to exposure to harmful tests and imaging. When in doubt, the UPT test should always be repeated with urine dilution and results should be confirmed.
